# Causal associations between psoriasis and atopic dermatitis: A bidirectional Mendelian randomization study

**DOI:** 10.1371/journal.pone.0322602

**Published:** 2025-09-08

**Authors:** Zixia Wang, Hao Nie, Xinyu Fan, Muyao Wang, Weili Chen, Quncai Huang, Junchao Xiao, Wenhui Wang

**Affiliations:** 1 Dongguan TCM Hospital of Guangzhou University of Chinese Medicine, Dongguan, China; 2 Guangzhou University of Chinese Medicine, Guangzhou, China; 3 Guangdong University of Technology, Guangzhou, China; Universita degli Studi di Roma Tor Vergata, ITALY

## Abstract

**Background:**

Although previous studies suggested associations between psoriasis and atopic dermatitis (AD), the directionality and causality of these relationships remain controversial. This study employed bidirectional Mendelian randomization to investigate the potential causal relationships between these two inflammatory skin conditions.

**Methods:**

Genome-wide association statistics were obtained for psoriasis and AD from large-scale consortia and meta-analyses of genome-wide association studies. Inverse-variance weighting, as the primary analysis, was combined with five complementary sensitivity analyses to evaluate the robustness and potential pleiotropy of the data. Additionally, we performed gene mapping of psoriasis-associated single-nucleotide polymorphisms and subsequent pathway analysis to further elucidate the potential relationships.

**Results:**

Genetic predisposition to psoriasis was significantly associated with a decreased risk of AD (odds ratio = 0.876; 95% confidence interval = 0.834–0.921; *p *= 1.6 × 10^−7^). Conversely, genetic predisposition to AD did not affect the risk of psoriasis. The associations remained consistent across multiple sensitivity analyses, and no evidence of horizontal pleiotropy was observed. Gene mapping identified eight key genes (*ENSG00000249738*, *ENSG00000291336*, *ENSG00000291338*, *ENSG00000285703*, *OR2W1-AS1*, *HLA-DQA1*, *FBXL18*, and nitric oxide synthase 2 (*NOS2*) located on chromosomes 5, 6, 7 and 17. Notably, *NOS2* emerged as a core gene involved in key biological processes, including the TCR signaling pathway and protein metabolism.

**Conclusions:**

This comprehensive MR study provided evidence of the protective causal effect of psoriasis on the risk of AD, whereas no reverse causal relationship was noted. These findings enhanced our understanding of the relationship between psoriasis and Ad and identified potential implications for their clinical management.

## Introduction

Psoriasis is a common debilitating disease of the skin and/or joints. Approximately 90% of all cases involve chronic plaque psoriasis, also termed psoriasis vulgaris, making it the most prevalent of the four types of the disease [1]. Chronic plaque psoriasis manifests as thick patches of erythematous skin with overlying white or silvery scales, with patients regularly experiencing symptoms including pain, itching, and bleeding [2]. Atopic dermatitis (AD), also called eczema, is a chronic autoimmune pruritic inflammatory skin condition that affects skin creases and flexure surfaces. Psoriasis and AD are the two most prevalent chronic inflammatory skin conditions globally, and both diseases are characterized by immune dysfunction and inflammatory cell infiltration in skin lesions [3]. Multifactorial interactions between hereditary and environmental factors are the main causes of both diseases, and these diseases markedly decrease the quality of life and emotional well-being of patients, in addition to various comorbidities closely related to their occurrence.

Psoriasis and atopic dermatitis (AD) represent two distinct chronic inflammatory skin conditions that share some clinical manifestations but differ fundamentally in their pathogenic mechanisms [4]. While both conditions present with erythema and pruritus, their cutaneous lesions exhibit distinct morphological and distributional patterns [5]. Psoriasis typically manifests as well-demarcated, thick, scaly plaques predominantly affecting extensor surfaces, whereas AD characteristically presents with poorly defined, excoriated patches and plaques in flexural areas. These clinical distinctions reflect underlying differences in their immunological signatures, with psoriasis being predominantly driven by Th1/Th17 responses and AD by Th2-mediated inflammation [6]. Despite their apparent differences, the potential interplay between these conditions has intrigued researchers, particularly given their shared inflammatory nature and occasional clinical overlap.

Previous observational studies reported both the coexistence and mutual exclusivity of psoriasis and AD, with both diseases caused by T cells activated by specific antigens [4,7,8]. Ivert *et al.*[9] found that AD was related to several autoimmune illnesses, especially those involving the skin such as psoriasis, with stronger relationships being reported for individuals with multiple autoimmune comorbidities [10]. However, some studies suggested that psoriasis and AD can coexist, although no clear consensus has been reached, making the causal direction between the risks of these diseases unclear [11].

Conventional observational epidemiological studies on psoriasis and AD face several challenges in elucidating disease etiology and inferring causality, such as the effects of potential confounders and the presence of reverse causality bias [12]. Therefore, more reliable methods are urgently needed to eliminate these interfering factors and confirm the potential causal relationships between psoriasis and AD. Mendelian randomization (MR), which has recently garnered increasing attention, utilizes genetic variants as the instrumental variables (IVs). MR is an effective strategy for determining the causal effects of exposure to genetic variants with single-nucleotide polymorphisms (SNPs) on outcomes independent of residual reverse or confounding causality bias [13]. MR has three core assumptions: genetic variants should exhibit a robust association with exposure; the association between the genetic variants and exposure should be independent of the potential confounders; and the influence of the genetic variants on the risk of the outcome should only occur through a risk factor without any potential effect from alternative pathways.

This study represents a significant addition to understanding of the causal relationship between psoriasis and atopic AD by employing bidirectional Mendelian Randomization (MR)—a methodological innovation that resolves longstanding controversies regarding the directionality and causality of their association. Unlike previous studies that predominantly relied on observational data, this research leverages genetic instruments to infer causality, effectively minimizing biases from confounding factors and reverse causation. Besides, this study could improve our understanding of the pathogenesis of AD, especially in the context of immune dysfunction.

## Materials and methods

### Study design

**Fig 1:**
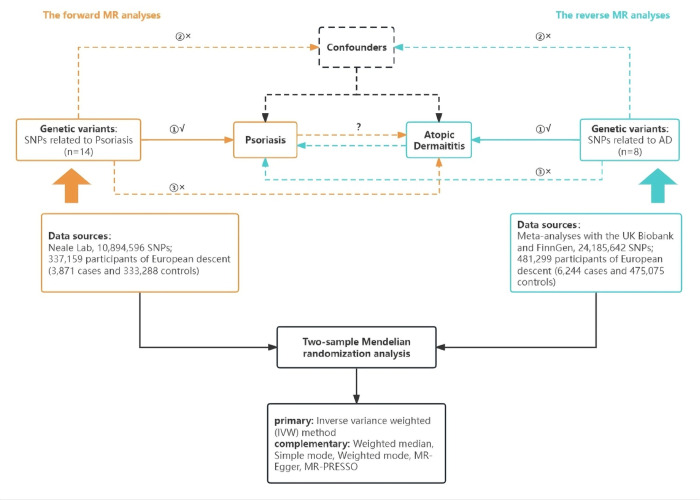
Overview of the study design. The orange boxes and arrows represent the forward MR analyses, with psoriasis as the exposure and AD as the outcome, while the cyan boxes and arrows represent the reverse MR analyses, with AD as the exposure and psoriasis as the outcome. Abbreviations: MR, Mendelian randomization; AD, atopic dermatitis; SNPs, single-nucleotide polymorphisms.

All of the genome-wide association statistics obtained from the corresponding consortia are publicly available. The flowchart of the study design is presented in Fig 1.

### Data sources

The genome-wide association statistics for psoriasis and AD used in this study were both obtained from genome-wide association studies (GWASs), which were limited to research that only included individuals of European ancestry to prevent population stratification bias [14]. Publicly available summary statistics (β, standard error, and p-value) for psoriasis reported by the Neale laboratory in the IEU Open GWAS project (ID:ukb-a-100) were assessed. These data included 3871 patients with psoriasis and 333,288 controls in the UK Biobank. The summary-level data for AD (ID: ebi-a-GCST90018784) in our study were obtained from a cross-population atlas study conducted by Sakaue *et al.*[15]. The study also incorporated data from the GWAS Catalog, which included 24,185,642 SNPs across 481,299 participants (6224 patients and 475,075 controls) of European ancestry. The main characteristics of these GWAS data are presented in Table 1.

**Table 1:** Primary information of the utilized consortia and data.

Phenotypes	GWAS ID	Sample size	SNPs	Author	Consortium	Link	Year
Psoriasis	ukb-a-100	337,159	10,894,596	Neale	Neale Lab	https://gwas.mrcieu.ac.uk/datasets/ukb-b-6134/	2017
Atopic dermatitis	ebi-a-GCST90018784	481,299	24,185,642	Saori Sakaue	NA	https://gwas.mrcieu.ac.uk/datasets/ebi-a-GCST90018784/	2021

### Selection of genetic instruments

For selection, the IVs required a threshold of genome-wide significance (*P *< 5 × 10^−8^) to ensure a robust association between the SNPs and exposure. We performed linkage disequilibrium clumping to include independent SNPs (r^2^ < 0.001; clumping window size = 1000 kb) [16]. Palindromic SNPs with intermediate allele frequencies were excluded from the IVs. To detect pleiotropic pathways, the PhenoScanner database was searched for correlations between specific genetic variants such as IVs and other phenotypes. Specific examples were phenotypes associated with inflammatory bowel disease or body mass index (BMI), which are recognized as risk factors for AD [17,18]. Variants correlated with these and other phenotypes were classified as confounders and excluded from MR.

### Statistical analysis

The study employed bidirectional two-sample MR to examine the cause-and-effect relationships between psoriasis and AD. The principal statistical method was inverse-variance weighting (IVW), which was complemented by five sensitivity analyses: weighted median, simple mode, weighted mode, MR-Egger regression, and MR Pleiotropy RESidual Sum and Outlier (MR-PRESSO). These six approaches identified IVs with horizontal pleiotropy effects based on different assumptions. IVW is characterized by the assumption that all included SNPs are effective genetic instruments, and a comprehensive process of Wald estimates and meta-analysis was used to evaluate the overall effect of exposure on outcome [19]. In circumstances in which horizontal pleiotropy and heterogeneity were absent, the IVW result was considered the most reliable estimation [20]. In addition, the weighted median method generates a better casual estimate when >50% of the instruments are derived from valid IVs [21]. Although less powerful than IVW, simple mode is recognized as a robust method for detecting pleiotropy [22]. Based on the instrument strength being independent of the direct effect (InSIDE) hypothesis, MR-Egger regression facilitates judgment of the potential impact of pleiotropy on the intercept. Specifically, an intercept of zero indicates the absence of horizontal pleiotropy. When the InSIDE assumption is violated, the weighted mode estimate technique outperforms MR-Egger regression for detecting causal effects, as indicated by lower type I error rates and smaller deviation [23]. The weighted mode estimation method can detect causal effects. Compared with MR-Egger regression, it has less deviation and a lower type I error rate when the InSIDE hypothesis is violated [23]. MR-PRESSO can detect significant outliers and statistics including MR-Egger regression and horizontal pleiotropy effects after correction to remove corresponding outliers [24].

Cochrane’s Q value was used to further determine the heterogeneity among all SNPs, and “leave-one-out” analysis was conducted to determine whether the results were influenced by any independent potentially heterogeneous SNPs. Further, we calculated the F-statistic separately to quantify the strength of combined SNPs and each individual SNP. To classify the phenotypic variations, F-value > 10 for the corresponding SNP(s) was considered sufficiently strong, whereas F-value ≤ 10 denoted a weak instrument [25]. Finally, we performed reverse MR analyses of psoriasis and AD that were similar to those of the forward MR analyses.

All analyses were performed with R software version 4.2.2 using the TwoSampleMR (version 0.5.6) and MR-PRESSO (version 1.0) packages.

### SNP-to-gene mapping and pathway analysis

To further investigate the potential biological mechanisms underlying our findings, we utilized g:Profiler online tools (https://biit.cs.ut.ee/gprofiler/gost) to match all harmonized psoriasis-associated SNPs to the corresponding genes. The specific positions of identifiable genes were then mapped on the chromosome image using the “Rcircos” package in R software. In addition, we identified the signaling pathways involving nitric oxide synthase 2 (*NOS2,* located on 17q11.2), the identified core gene, through the Reactome (https://reactome.org/) and performed pathway analysis.

## Results

### Directional relationship from psoriasis to AD

**Fig 2:**
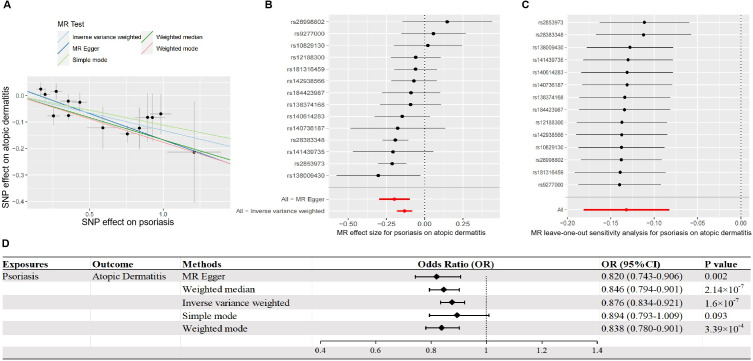
MR to identify the causal impact of genetically predicted psoriasis on the risk of AD. **(A)** Scatter plots of the genetic effects of IVs on the genetic predisposition to psoriasis and the subsequent risk of AD. The slope of the straight lines with different colors indicates the causal estimate in the corresponding methods of MR. Each black dot corresponds to an IV included in MR, whereas the gray bars represent the SDs of the genetic effect estimations. **(B)** Forest map of the visualized causal influences of each included SNP on the risk of AD risk. **(C)** “Leave-one-out” plots of the causal relationship between psoriasis and AD. **(D)** Causal effect of psoriasis on AD visualized in a forest map using five methods. Abbreviations: AD, atopic dermatitis; MR, Mendelian randomization; IVs, instrument variables; SD, standard deviation.

To investigate the causal influence of psoriasis on the risk of AD, we identified 14 independent SNPs as IVs in psoriasis. The detailed characteristics of these IVs are listed in S1 Table. As the primary method, IVW suggested a significant causal relationship between psoriasis and AD. Specifically, each one standard deviation (SD) decrease in psoriasis morbidity was associated with an approximately 12.4% higher risk of developing AD (odds ratio [OR] = 0.876, 95% confidence interval [CI] = 0.834–0.921, *p *= 1.6 × 10^−7^; Fig 2A, 2D).

The robustness of this finding was supported by consistent results across three other estimation methods (Fig 2D), namely MR Egger (OR = 0.820; 95% CI = 0.743–0.906; *p *= 0.002), weighted median (OR = 0.846; 95% CI = 0.794–0.901; *p *= 2.14 × 10^−7^), and weighted mode (OR = 0.838; 95% CI = 0.780–0.901; *p *= 3.39 × 10^−4^). As presented in S2 Table, comprehensive sensitivity analyses revealed no heterogeneity among these IVs in both the Cochrane’s Q test (*P*_Q_ > 0.05) and the MR-Egger test (*P*_intercept_ > 0.05) and no horizontal pleiotropy in the MR-PRESSO global test (P_global_ > 0.05). Additionally, no noticeable alterations in the estimated causal effect were detected in individual SNP removed in the “leave-one-out” analysis (Fig 2B–2C).

The combined F-statistic of the included 14 SNPs was 12.8, indicating sufficient instrument strength. However, the F-values of the individual SNP varied considerably (range: 0.9–179.3), suggesting that the individual SNPs had varied strengths of association with psoriasis. We therefore conducted a subgroup analysis and categorized the 14 SNPs as strong or weak instruments. Importantly, the causal association remained consistent in direction and significance in both subgroups (S3–S4 Tables), further supporting the reliability and robustness of our MR findings.

### SNP-to-gene mapping and pathways analysis

**Fig 3:**
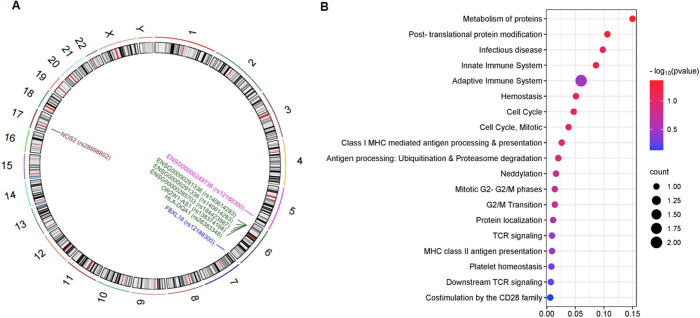
SNP-mapped genes and *NOS2*-related molecular pathways. (A) SNP-to-gene mapping displaying the genes associated with the forward SNPs, both of which are labeled in the figure. (B) Pathway analysis demonstrating the biological mechanism related to the *NOS2* gene. Abbreviations: SNP, single-nucleotide polymorphism; *NOS2*, nitric oxide synthase 2.

In this study, we mapped the casual SNPs to genes to identify the genes potentially influenced by SNPs associated with both psoriasis and AD. In the aggregate, the 14 SNPs were detected in eight genes located on chromosomes 5, 6, 7, and 17, namely *ENSG00000249738* (5q33.3), *ENSG00000291336* (6p22.2), *ENSG00000291338* (6p 22.2), *ENSG00000285703* (6p22.1), *OR2W1-AS1* (6p22.1), *HLA-DQA1* (6p21.32), *FBXL18* (7p22.1), and *NOS2* (Fig 3A). Based on previous research [26,27], we recognized *NOS2* as the core gene because of its close connections to both psoriasis and AD. The findings of pathway analysis illustrated the significant enrichment of *NOS2* in several biological processes, including protein metabolism, post-translational protein modification, neddylation, and protein localization. The findings also indicated that *NOS2* is involved in several immune-related processes such as class I MHC-mediated antigen processing and presentation, antigen processing through ubiquitination and proteasome degradation, and costimulation by the CD28 family (Fig 3B).

### Directional relationship from AD to psoriasis

The comprehensive meta-analysis of large-scale GWASs identified eight SNPs with robust associations with AD. Detailed information on each SNP is provided in Summary Table 5. The total strength (F-statistic) of the integrated eight SNPs was 14.9, with the F-values of the individual SNPs ranging 10.1–28.7. These results indicated that either the incorporated SNPs or each separate SNP had sufficient strength to explain the phenotypic variations of AD.

**Fig 4:**
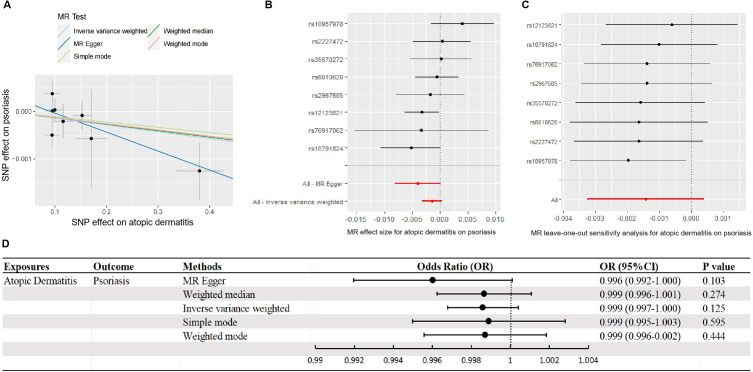
MR of the causal estimates of the effects of genetic predisposition to AD on the risk of psoriasis. (A) Scatter plots of the effects of IVs on the genetic predisposition to AD and the subsequent risk of psoriasis. The slope of the straight lines in different colors indicates the causal estimation in the corresponding methods of MR. Each black dot corresponds to an IV included in MR, whereas the gray bars represent the SD of the estimation of the genetic effect. (B) Forest map of the visualized causal influence of each included SNP on the risk of psoriasis. (C) “Leave-one-out” plots of the causality between AD and psoriasis. (D) Causal impact of AD on the risk of psoriasis visualized as a forest map of the five methods. Abbreviations: MR, Mendelian randomization; AD, atopic dermatitis; IVs, instrument variables; SD, standard deviation.

As detailed in Fig 4D, no evidence providing a positive or reverse causal relationship between genetic predisposition to AD and the risk of psoriasis was obtained according to the results of IVW, MR-Egger, weighted median, simple mode, and weighted mode using the full set of eight SNPs (*p *> 0.05). Coincidently, no outlying SNP was identified by the outlier-corrected MR-PRESSO method (causal estimate = −0.0014, SD = −0.0009, T-statistic = −1.5356, *p* = 0.1685; S6 Table). No noticeable heterogeneity or pleiotropic effects for the MR estimate of the causal effect of AD on psoriasis were detected using either the MR-Egger, Cochrane’s Q, or MR-PRESSO global tests (all *p* *> *0.05; Fig 4A–4B, S6 Table). The “leave-one-out” analysis also identified no significant alterations in the MR estimates when each specific SNP was discarded or returned (Fig 4C). This finding confirmed the robustness and effectiveness of our results.

## Discussion

To the best of our knowledge, this is the first study to investigate the reciprocal impact between psoriasis and AD. This study identified a significant causal relationship between increased psoriasis morbidity (genetically proxied) and a decreased risk of AD (genetically proxied), whereas a genetic predisposition to AD did not influence the later risk of psoriasis.

In an earlier epidemiological study of a European population, we reported that individuals with psoriasis had a 25-fold lower prevalence of AD [28]. An observational study involving 29,159 German patients by Christophers *et al.*[29] confirmed this finding and suggested that the concurrence of psoriasis and AD was extremely rare. Another cross-sectional study also recorded a lower prevalence of AD in individuals with psoriasis than in healthy controls [30]. The findings of this MR study were consistent with those of previous studies, supporting the existence of a causal effect of psoriasis on AD. Nonetheless, based on the shared immunopathogenesis of psoriasis and AD, the accumulated clinical studies suggest that the two diseases can coexist. For example, a retrospective review by Barry *et al.*[11] found that the rate of concomitant AD and psoriasis was 1.5% with notable hand involvement. A recent nationwide cohort study in Korea also reported a bidirectional association between psoriasis and AD [31]. These findings contradict the results of our study, which found that psoriasis was closely correlated with a lower risk of AD. Because of these inconsistent findings, the causal relationship between psoriasis and AD remains to be confirmed.

Our findings of a protective effect of psoriasis on AD risk align well with the distinct immunological profiles of these conditions. The inverse relationship we observed may be explained by the antagonistic nature of their predominant T-helper cell responses. The Th1/Th17-dominated environment in psoriasis might suppress the development of Th2-driven allergic inflammation characteristic of AD. This molecular antagonism is reflected in their contrasting clinical presentations: while both conditions manifest with inflammation and pruritus, psoriatic lesions typically present as well-defined, thick, erythematous plaques with silvery scales, predominantly affecting extensor surfaces. In contrast, AD lesions are generally poorly demarcated, with a predilection for flexural areas and accompanied by intense pruritus leading to excoriation. These distinct clinical and immunological features support our genetic findings and suggest that the pathogenic mechanisms underlying psoriasis may actually protect against the development of AD through complex immunological interactions.

Our gene mapping analysis revealed several key genes that provide mechanistic insights into the relationship between psoriasis and AD. Most notably, *NOS2* emerged as a core gene, showing elevated expression in psoriatic lesions while maintaining lower levels in AD-affected skin, which aligns with our observed protective effect of psoriasis on AD risk [32]. The identification of *HLA-DQA1* is particularly significant, as its variants demonstrate distinct immunological effects in these conditions. While psoriasis-associated variants promote Th1/Th17 responses, AD-linked variants predominantly drive Th2-mediated inflammation [10]. This differential immune programming may explain the inverse relationship we observed between these conditions. Our pathway analysis revealed that several identified genes, including *FBXL18* and novel transcripts (*ENSG00000249738*, *ENSG00000291336*), are involved in protein metabolism and TCR signaling pathways. These pathways are crucial in determining T-cell fate and function, potentially explaining the antagonistic relationship between psoriasis and AD [33]. Additionally, the identification of *OR2W1-AS1*, located near the HLA complex, suggests potential regulatory functions in immune responses, though further functional studies are needed to elucidate its precise role. Indeed, there is evidence of directional associations between psoriasis and AD, such as their shared immune pathways and histopathological features [34]. Both diseases are characterized by activated T cell infiltration in the skin accompanied by enhanced keratinocyte proliferation, resulting in skin thickening [35]. In addition, the immune pathways of psoriasis and AD both involve Th1 and Th22 cells, with Th17 cells specifically present in the pediatric, intrinsic, and Asian types of both conditions [35–37]. However, the development of psoriatic lesions is mainly attributed to chronic inflammation through the TNFα/interleukin (IL)-17/Il-23/Th17 axis, whereas AD is associated with skewed correlations among Th2 cells, IL-4, and IL-13 [35,38,39]. In psoriasis, damaged keratinocytes release antimicrobial peptides such as β-defensins and LL-37 in the initiation phase, resulting in the formation of LL-37–DNA complexes that further amplify Toll-like receptor 9 signaling to activate plasmacytoid dendritic cells (pDCs) [40]. These activated pDCs produce interferons (IFNs), including IFNα and IFNβ, which subsequently expedite the maturation of myeloid dendritic cells (mDCs). MDCs in turn release IL-12, IL-23, and TNF-α to stimulate Th1 and Th17 cells [36,41]. IFNα is also involved in the secretion of IFNγ and IL-17 and the differentiation of Th1 and Th17 cells. Generally, the IL-17 family consists of six members (IL17A–F), among which IL-17A displays a strong correlation with the pathogenesis of psoriasis [42]. Other members of the IL-17 family, such as IL-17C and IL-17F, are expressed prominently in psoriasiform dermatitis, and they enhance innate defenses in epithelial cells [43]. IL-23 dominates in plaque psoriasis and plays a major role in modulating JAK2/TYK2 and STAT signaling, thereby increasing IL-17 production by immune cells [42,44,45]. Taken together, these findings demonstrate that the central mechanism of psoriasis is related closely to adaptive immune pathways comprising IL-17 and IL-23, together with the pivotal roles of Th17 cells and TNF-α.

**Fig 5:**
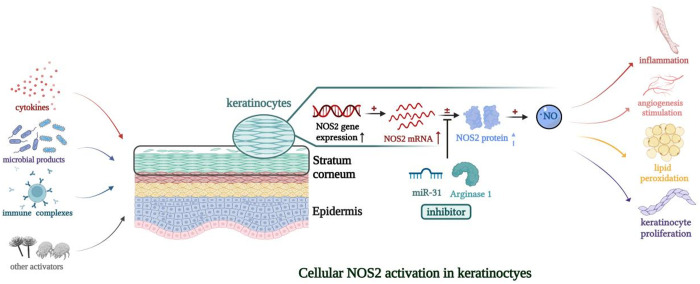
A possible model of the mechanism linking *NOS2* expression to the development of psoriasis. A variety of activators such as cytokines, microbial products, and immune complexes, stimulate cellular *NOS2* expression in keratinocytes. The formed •NO then induces inflammation, angiogenesis stimulation, lipid peroxidation, keratinocyte proliferation, and other effects through interactions with other superoxide moieties. In this process, *NOS2* protein expression is not fully correlated with its mRNA expression because *NOS2* protein translation can be inhibited by various factors (*e.g.*, miR-31, arginase 1).

Among the genes identified in this study, *NOS2* is considered the core gene because of its strong associations with both diseases. According to previous genome-wide association analyses, *NOS2* has been postulated to be a susceptibility gene in psoriasis based on its increased expression [46,47]. Pathological manifestations in patients with psoriasis, including inflammation and vascular and keratinocyte proliferation, are caused by cellular *NOS2* activation (Fig 5). In contrast to low *NOS2* expression in AD lesions, Köhler *et al.* identified significantly increased *NOS2* mRNA expression in psoriatic epidermis and proved that *NOS2*-derived low levels of NO mediated downstream psoriatic effects of IL-17 [48]. Recently, *NOS2*/*iNOS (*inducible nitric oxide synthase) was proposed as a novel gene classifier to diagnose psoriasis and AD with 100% accuracy by quantitative PCR [49]. Other articles similarly recognized the potential of *NOS2* as a specific molecular disease classifier for both diseases [26,27,50].

Regarding the strong genetic backgrounds of psoriasis and AD, several different chromosomal loci have been identified in each disease with minimal overlap [34]. Studies have implied that HLA-C*06:02 (on PSORS1 6p21.33) is a major susceptibility gene locus for psoriasis, whereas null mutations of the *FLG* gene (1q21.3) have been suggested to increase the risk of AD [5,36,41,51]. Particularly in European populations, *HLA-C*06:02 demonstrates a strong association with psoriasis, with an odds ratio of approximately 3.5, while *FLG* null mutations are found in up to 50% of moderate-to-severe AD patients of European descent [52]. Current genome-wide comparative analyses have confirmed the presence of *FLG* gene mutations in the epidermal differentiation complex on chromosome 1q21.3 in AD but not in psoriasis [10,53]. Similarly, removal of the late cornified envelope genes 3B/3C on chromosome 1q21 has been linked to psoriasis but not AD [10,54]. Nevertheless, other *FLG* genetic variants have been confirmed to confer susceptibility to psoriasis, especially in the Chinese population, indicating a shared region on the genome between AD and psoriasis [55,56]. In general, the majority of genetic analyses supported the mutual exclusivity of psoriasis and AD, although several overlapping loci have been discovered recently [10,57,58].

### Strengths and limitations of the study

The study had several strengths. The major advantage was its bidirectional MR design, which strengthened the causal inference between psoriasis and AD. The bidirectional analysis was also used to determine the existence of a two-way association between the diseases when compared to standard MR analysis. In addition, the data utilized in this study were derived from consortia with adequate sample sizes, with the identified SNPs divided into two groups according to the strength of statistical power, thereby ensuring the robustness of our findings. Moreover, we detected and excluded potential outliers after implementing MR-PRESSO and MR-Egger regression intercept tests, which contributed to low heterogeneity and bias in our study. Ultimately, no duplicate SNP was detected in the GWAS summary data related to psoriasis and AD.

However, some limitations requiring further consideration must be acknowledged. First, the conflicting results between our study and other studies might reflect the influence of unmeasured potential confounding factors on causal inferences. Second, the data used in our study were exclusively related to individuals of European ancestry, thereby constraining the generalizability of our findings to other demographic cohorts. For example, studies have identified specific genetic and manifestation characteristics of psoriasis or AD in Asian patients, distinguishing them from other ethnic groups [4,34]. In addition, the depth of our study was constrained because of the lack of detailed classified data, precluding hierarchical analyses to further investigate the causal associations between different subtypes of the two diseases, such as the four subtypes of psoriasis (psoriasis vulgaris, erythrodermic psoriasis, pustular psoriasis, and arthritic psoriasis). Although no pleiotropic effect was detected in our analysis, we could not dismiss the possibility that genetic instruments proxied for psoriasis and AD were related to other traits that could affect the final outcome. Furthermore, the negative association between psoriasis and AD might have been induced by overlapping genes rather than a causal link. However, the limited shared genetic background between psoriasis and AD minimized the potential of this bias [34].

## Conclusion

This study identified a causal association between genetically predicted psoriasis and a lower risk of AD, whereas the hypothesis of reverse causality was not confirmed. However, we cannot dismiss the possibility that AD influences the incidence and progression of psoriasis. Further experimental and clinical studies are warranted to validate and extend our findings and discover the molecular mechanisms underlying the observed causal associations.

## Supporting information

S1 Table Instrumental variables used in MR analysis of the association between psoriasis and AD. Detailed information of SNPs used in MR analysis: From psoriasis to AD.
(XLSX)

S2 Table Detailed information of the sensitive analyses used in MR analysis of the association between psoriasis and AD. Cochran’s Q test, MR Egger test and MR PRESSO global test used in this study: From psoriasis to AD.
(XLSX)

S3 Table Strong instrumental variables correlated sensitive analyses used in MR analysis of the association between psoriasis and AD. Detailed information of strong instruments group in subgroup analyses: From psoriasis to AD.
(XLSX)

S4 Table Weak instrumental variables correlated sensitive analyses used in MR analysis of the association between psoriasis and AD Detailed information of weak instruments group in subgroup analyses: From psoriasis to AD.
(XLSX)

S5 Table Instrumental variables used in MR analysis of the association between AD and psoriasis. Detailed information of SNPs used in MR analysis: From AD to psoriasis.
(XLSX)

S6 Table Detailed information of the sensitive analyses used in MR analysis of the association between AD and psoriasis. Cochran’s Q test, MR Egger test and MR PRESSO global test used in this study: From AD to psoriasis.
(XLSX)
